# 6,7-Dihydro-4-(4-methoxy­phen­yl)-3-methyl-6-oxo-1-phenyl-1*H*-pyrazolo[3,4-*b*]pyridine-5-carbonitrile

**DOI:** 10.1107/S1600536808027852

**Published:** 2008-09-06

**Authors:** Xin-Ying Zhang, Xiao-Yan Li, Xia Wang, Dong-Fang Li, Xue-Sen Fan

**Affiliations:** a School of Chemical and Environmental Sciences, Henan Key Laboratory for Environmental Pollution Control, Henan Normal University, Xinxiang, Henan 453007, People’s Republic of China

## Abstract

In the title compound, C_21_H_16_N_4_O_2_, the dihedral angle between the meth­oxy-substituted benzene ring and the ring system formed by the pyridinone ring and the pyrazole ring is 57.4 (1)°, and that between the unsubstituted phenyl ring and the ring system is 135.6 (1)°. In the crystal structure, mol­ecules are linked together *via* inter­molecular N—H⋯O hydrogen bonds.

## Related literature

For the biological and pharmacological activities of pyrazolo[3,4-*b*]pyridine derivatives, see Falcó *et al.* (2005 ▶); Ludwig *et al.* (2004 ▶). For a related structure, see Quiroga *et al.* (1999 ▶).
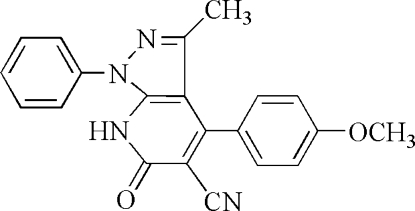


## Experimental

### 

#### Crystal data

C_21_H_16_N_4_O_2_
*M* *_r_* = 356.38
Triclinic, 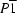

*a* = 7.0621 (11) Å
*b* = 11.0272 (17) Å
*c* = 12.1743 (19) Å
α = 68.467 (2)°
β = 78.949 (2)°
γ = 87.471 (2)°
*V* = 865.2 (2) Å^3^
*Z* = 2
Mo *K*α radiation
μ = 0.09 mm^−1^
*T* = 295 (2) K
0.43 × 0.30 × 0.11 mm

#### Data collection

Bruker SMART CCD area-detector diffractometer
Absorption correction: none
6198 measured reflections
3136 independent reflections
2236 reflections with *I* > 2σ(*I*)
*R*_int_ = 0.019

#### Refinement

*R*[*F* ^2^ > 2σ(*F* ^2^)] = 0.042
*wR*(*F* ^2^) = 0.114
*S* = 1.02
3136 reflections
246 parameters
H-atom parameters constrained
Δρ_max_ = 0.15 e Å^−3^
Δρ_min_ = −0.21 e Å^−3^

### 

Data collection: *SMART* (Bruker, 1997 ▶); cell refinement: *SAINT* (Bruker, 1997 ▶); data reduction: *SAINT* (Bruker, 1997 ▶); program(s) used to solve structure: *SHELXTL* (Sheldrick, 2008 ▶); program(s) used to refine structure: *SHELXTL* (Sheldrick, 2008); molecular graphics: *SHELXTL* (Sheldrick, 2008); software used to prepare material for publication: *SHELXTL* (Sheldrick, 2008).

## Supplementary Material

Crystal structure: contains datablocks I, global. DOI: 10.1107/S1600536808027852/xu2451sup1.cif

Structure factors: contains datablocks I. DOI: 10.1107/S1600536808027852/xu2451Isup2.hkl

Additional supplementary materials:  crystallographic information; 3D view; checkCIF report

## Figures and Tables

**Table 1:** Hydrogen-bond geometry (Å, °)

*D*—H⋯*A*	*D*—H	H⋯*A*	*D*⋯*A*	*D*—H⋯*A*
N1—H1⋯O1^i^	0.86	2.06	2.8523 (18)	153

Symmetry code: (i) 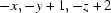.

**Fig. 1.**
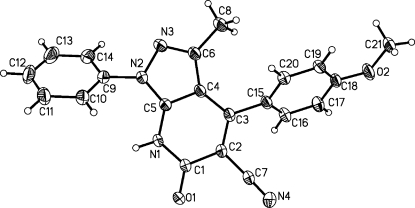
Molecular structure of the title compound with displacement ellipsoids drawn at the 30% probability level.

**Fig. 2.**
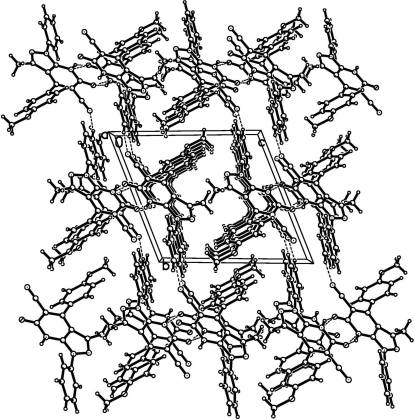
Crystal Structure of the title compound with view along the a axis. Intermolecular N—H···O hydrogen bonding is shown as dashed lines.

## supplementary crystallographic information

### Comment

Pyrazolo[3,4-*b*]pyridine derivatives have been found of interests for their biological and pharmacological activities, such as antiviral (Ludwig *et al.*, 2004). Moreover, pyrazolo[3,4-*b*]pyridin-6-ones as a subunit of pyrazolo[3,4-*b*]pyridine acted as potential hypnotic drugs in many cases (Falcó *et al.*, 2005). Due to their importance, many methods have been reported for the construction of pyrazolo[3,4-*b*]pyridine derivatives including pyrazolo[3,4-*b*]pyridin-6-ones (Quiroga *et al.*, 1999; Falcó *et al.*, 2005). Herein, we report the crystal structure of the title compound, one of pyrazolo[3,4-*b*]pyridin-6-one derivatives.

In the title compound there are four rings including two phenyl rings, one pyridinone ring and one pyrazole ring. The pyridinone ring and the pyrazole ring is almost co-planar and formed a ring system. The dihedral angle between this ring system and the methoxy-substituted phenyl ring is 57.4 (1)°, which is probably due to the repulsion of the cyano group on the pyridinone ring and the hydrogen atoms on the *ortho*-positions of the phenyl ring connected with the pyridinone ring, and the repulsion between these hydrogen atoms and the methyl group on the pyrazole ring. The dihedral angle between the non-substituted phenyl ring and the ring system is 135.6 (1)°.

In the crystal structure the molecules are connected *via* intermolecular N—H···O hydrogen bonding (Table 1).

### Experimental

To 1 ml of 1-butyl-3-methylimidazolium tetrafluoroborate ([bmim][BF_4_]) were added 4-methoxybenzaldehyde (1 mmol, II) and ethyl cyanoacetate (1 mmol). The mixture was stirred at 80 °C until the disappearance of II. Then, 5-amino-3-methyl-1-phenylpyrazole (1 mmol) and FeCl_3_.6H_2_O (0.2 mmol) was added and the mixture was continued to be stirred at the same temperature to complete the reaction (monitored by TLC). The reaction time was 10 h totally. Upon completion, the mixture was cooled to room temperature and 2 ml of 50% ethanol in water was added. The product was collected by suction and rinsed with water and cool ethanol in a yield of 90% as white solid. Single crystals of the title compound were obtained by slow evaporation of the solvent from an ethyl acetate-ethanol (1:1 *v/v*) solution.

### Refinement

H-atoms were included in calculated positions and treated as riding atoms with N—H = 0.86 Å and C—H = 0.93–0.96 Å, U_iso_(H) = 1.5U_eq_(C) for methyl and 1.2U_eq_(N,C) for others.

### Crystal data

**Table:**

C_21_H_16_N_4_O_2_	*Z* = 2
*M**_r_* = 356.38	*F*(000) = 372
Triclinic, *P*1	*D*_x_ = 1.368 Mg m^−^^3^
Hall symbol: -P 1	Mo *K*α radiation, λ = 0.71073 Å
*a* = 7.0621 (11) Å	Cell parameters from 1698 reflections
*b* = 11.0272 (17) Å	θ = 3.2–26.3°
*c* = 12.1743 (19) Å	µ = 0.09 mm^−^^1^
α = 68.467 (2)°	*T* = 295 K
β = 78.949 (2)°	Block, colourless
γ = 87.471 (2)°	0.43 × 0.30 × 0.11 mm
*V* = 865.2 (2) Å^3^	

### Data collection

**Table:**

Bruker SMART CCD area-detector diffractometer	2236 reflections with *I* > 2σ(*I*)
Radiation source: fine-focus sealed tube	*R*_int_ = 0.019
graphite	θ_max_ = 25.5°, θ_min_ = 2.9°
φ and ω scans	*h* = −8→8
6198 measured reflections	*k* = −13→13
3136 independent reflections	*l* = −14→14

### Refinement

**Table:**

Refinement on *F*^2^	Primary atom site location: structure-invariant direct methods
Least-squares matrix: full	Secondary atom site location: difference Fourier map
*R*[*F*^2^ > 2σ(*F*^2^)] = 0.042	Hydrogen site location: inferred from neighbouring sites
*wR*(*F*^2^) = 0.114	H-atom parameters constrained
*S* = 1.02	*w* = 1/[σ^2^(*F*_o_^2^) + (0.0514*P*)^2^ + 0.1601*P*] where *P* = (*F*_o_^2^ + 2*F*_c_^2^)/3
3136 reflections	(Δ/σ)_max_ < 0.001
246 parameters	Δρ_max_ = 0.15 e Å^−^^3^
0 restraints	Δρ_min_ = −0.21 e Å^−^^3^

### Special details

**Table:**

Geometry. All e.s.d.'s (except the e.s.d. in the dihedral angle between two l.s. planes) are estimated using the full covariance matrix. The cell e.s.d.'s are taken into account individually in the estimation of e.s.d.'s in distances, angles and torsion angles; correlations between e.s.d.'s in cell parameters are only used when they are defined by crystal symmetry. An approximate (isotropic) treatment of cell e.s.d.'s is used for estimating e.s.d.'s involving l.s. planes.
Refinement. Refinement of *F*^2^ against ALL reflections. The weighted *R*-factor *wR* and goodness of fit *S* are based on *F*^2^, conventional *R*-factors *R* are based on *F*, with *F* set to zero for negative *F*^2^. The threshold expression of *F*^2^ > σ(*F*^2^) is used only for calculating *R*-factors(gt) *etc*. and is not relevant to the choice of reflections for refinement. *R*-factors based on *F*^2^ are statistically about twice as large as those based on *F*, and *R*- factors based on ALL data will be even larger.

### Fractional atomic coordinates and isotropic or equivalent isotropic displacement parameters (Å^2^)

**Table:**

	*x*	*y*	*z*	*U*_iso_*/*U*_eq_	
C1	0.2756 (2)	0.56351 (17)	0.91520 (16)	0.0390 (4)	
C2	0.4579 (2)	0.63083 (17)	0.84855 (16)	0.0396 (4)	
C3	0.5717 (2)	0.60459 (16)	0.75386 (16)	0.0380 (4)	
C4	0.5056 (2)	0.50171 (17)	0.72613 (16)	0.0390 (4)	
C5	0.3325 (2)	0.43515 (16)	0.79290 (15)	0.0382 (4)	
C6	0.5776 (3)	0.43251 (18)	0.64820 (17)	0.0450 (5)	
C7	0.5055 (3)	0.7343 (2)	0.88358 (19)	0.0534 (5)	
C8	0.7609 (3)	0.4528 (2)	0.55762 (19)	0.0583 (6)	
H8A	0.7738	0.3832	0.5273	0.087*	
H8B	0.8686	0.4537	0.5950	0.087*	
H8C	0.7580	0.5345	0.4924	0.087*	
C9	0.1563 (3)	0.23971 (18)	0.79607 (16)	0.0457 (5)	
C10	−0.0341 (3)	0.2728 (2)	0.81733 (18)	0.0549 (5)	
H10	−0.0665	0.3585	0.8065	0.066*	
C11	−0.1762 (3)	0.1761 (3)	0.8550 (2)	0.0736 (7)	
H11	−0.3052	0.1966	0.8713	0.088*	
C12	−0.1284 (5)	0.0504 (3)	0.8685 (2)	0.0857 (9)	
H12	−0.2250	−0.0137	0.8923	0.103*	
C13	0.0630 (5)	0.0186 (2)	0.8470 (2)	0.0813 (8)	
H13	0.0947	−0.0670	0.8567	0.098*	
C14	0.2069 (3)	0.1128 (2)	0.81120 (19)	0.0623 (6)	
H14	0.3360	0.0915	0.7975	0.075*	
C15	0.7532 (2)	0.68056 (16)	0.68722 (16)	0.0386 (4)	
C16	0.8942 (3)	0.69110 (18)	0.74930 (17)	0.0455 (5)	
H16	0.8741	0.6498	0.8325	0.055*	
C17	1.0627 (3)	0.76181 (18)	0.68909 (17)	0.0490 (5)	
H17	1.1553	0.7678	0.7320	0.059*	
C18	1.0963 (2)	0.82417 (17)	0.56554 (17)	0.0437 (5)	
C19	0.9568 (3)	0.81699 (17)	0.50216 (17)	0.0452 (5)	
H19	0.9768	0.8600	0.4192	0.054*	
C20	0.7868 (3)	0.74526 (17)	0.56307 (16)	0.0429 (4)	
H20	0.6936	0.7404	0.5201	0.051*	
C21	1.3193 (3)	0.9516 (2)	0.39001 (19)	0.0655 (6)	
H21A	1.3223	0.8877	0.3534	0.098*	
H21B	1.4440	0.9940	0.3686	0.098*	
H21C	1.2248	1.0151	0.3623	0.098*	
N1	0.22050 (19)	0.46471 (13)	0.88333 (13)	0.0391 (4)	
H1	0.1144	0.4213	0.9209	0.047*	
N2	0.3060 (2)	0.33757 (15)	0.75584 (14)	0.0446 (4)	
N3	0.4599 (2)	0.33551 (16)	0.66572 (14)	0.0510 (4)	
N4	0.5383 (3)	0.8162 (2)	0.9136 (2)	0.0929 (8)	
O1	0.17078 (18)	0.59323 (13)	0.99468 (12)	0.0513 (4)	
O2	1.27013 (19)	0.88929 (14)	0.51707 (13)	0.0605 (4)	

### Atomic displacement parameters (Å^2^)

**Table:**

	*U*^11^	*U*^22^	*U*^33^	*U*^12^	*U*^13^	*U*^23^
C1	0.0338 (9)	0.0395 (10)	0.0403 (10)	−0.0069 (8)	0.0009 (8)	−0.0136 (8)
C2	0.0333 (9)	0.0393 (10)	0.0432 (10)	−0.0072 (8)	−0.0002 (8)	−0.0141 (8)
C3	0.0308 (9)	0.0356 (9)	0.0413 (10)	−0.0025 (7)	−0.0011 (8)	−0.0091 (8)
C4	0.0307 (9)	0.0399 (10)	0.0413 (10)	−0.0024 (7)	0.0026 (7)	−0.0131 (8)
C5	0.0343 (9)	0.0380 (10)	0.0401 (10)	−0.0019 (7)	0.0001 (8)	−0.0148 (8)
C6	0.0369 (10)	0.0442 (11)	0.0488 (11)	−0.0016 (8)	0.0053 (8)	−0.0173 (9)
C7	0.0392 (11)	0.0556 (13)	0.0624 (13)	−0.0165 (9)	0.0130 (9)	−0.0272 (11)
C8	0.0484 (12)	0.0595 (13)	0.0603 (13)	−0.0036 (10)	0.0143 (10)	−0.0260 (11)
C9	0.0490 (11)	0.0476 (11)	0.0413 (11)	−0.0137 (9)	0.0009 (9)	−0.0200 (9)
C10	0.0488 (12)	0.0673 (14)	0.0520 (12)	−0.0157 (10)	−0.0024 (9)	−0.0273 (11)
C11	0.0568 (14)	0.108 (2)	0.0591 (14)	−0.0353 (14)	0.0017 (11)	−0.0365 (15)
C12	0.105 (2)	0.095 (2)	0.0552 (15)	−0.0659 (18)	0.0019 (14)	−0.0253 (14)
C13	0.117 (2)	0.0580 (15)	0.0675 (16)	−0.0362 (15)	−0.0015 (15)	−0.0248 (13)
C14	0.0752 (15)	0.0500 (13)	0.0617 (14)	−0.0137 (11)	0.0039 (11)	−0.0269 (11)
C15	0.0301 (9)	0.0368 (10)	0.0430 (10)	−0.0029 (7)	0.0023 (8)	−0.0117 (8)
C16	0.0358 (10)	0.0496 (11)	0.0407 (10)	−0.0060 (8)	−0.0007 (8)	−0.0070 (9)
C17	0.0346 (10)	0.0534 (12)	0.0508 (12)	−0.0058 (9)	−0.0056 (9)	−0.0101 (10)
C18	0.0339 (10)	0.0387 (10)	0.0512 (12)	−0.0066 (8)	0.0069 (8)	−0.0144 (9)
C19	0.0470 (11)	0.0420 (10)	0.0377 (10)	−0.0051 (8)	0.0063 (8)	−0.0105 (8)
C20	0.0390 (10)	0.0443 (10)	0.0434 (11)	−0.0039 (8)	−0.0023 (8)	−0.0158 (9)
C21	0.0598 (14)	0.0550 (13)	0.0643 (15)	−0.0165 (10)	0.0248 (11)	−0.0179 (11)
N1	0.0316 (8)	0.0390 (8)	0.0424 (8)	−0.0108 (6)	0.0065 (6)	−0.0151 (7)
N2	0.0395 (8)	0.0440 (9)	0.0488 (9)	−0.0099 (7)	0.0068 (7)	−0.0215 (7)
N3	0.0469 (9)	0.0504 (10)	0.0520 (10)	−0.0053 (8)	0.0107 (8)	−0.0238 (8)
N4	0.0738 (14)	0.0961 (17)	0.1204 (19)	−0.0448 (12)	0.0337 (13)	−0.0735 (16)
O1	0.0405 (7)	0.0616 (9)	0.0534 (8)	−0.0166 (6)	0.0137 (6)	−0.0320 (7)
O2	0.0430 (8)	0.0640 (9)	0.0589 (9)	−0.0189 (7)	0.0111 (7)	−0.0125 (7)

### Geometric parameters (Å, °)

**Table:**

C1—O1	1.236 (2)	C11—H11	0.9300
C1—N1	1.377 (2)	C12—C13	1.381 (4)
C1—C2	1.452 (2)	C12—H12	0.9300
C2—C3	1.388 (2)	C13—C14	1.376 (3)
C2—C7	1.429 (3)	C13—H13	0.9300
C3—C4	1.417 (2)	C14—H14	0.9300
C3—C15	1.484 (2)	C15—C20	1.391 (2)
C4—C5	1.398 (2)	C15—C16	1.392 (2)
C4—C6	1.433 (2)	C16—C17	1.375 (2)
C5—N2	1.343 (2)	C16—H16	0.9300
C5—N1	1.361 (2)	C17—C18	1.382 (3)
C6—N3	1.313 (2)	C17—H17	0.9300
C6—C8	1.496 (2)	C18—O2	1.364 (2)
C7—N4	1.139 (2)	C18—C19	1.384 (3)
C8—H8A	0.9600	C19—C20	1.389 (2)
C8—H8B	0.9600	C19—H19	0.9300
C8—H8C	0.9600	C20—H20	0.9300
C9—C10	1.379 (3)	C21—O2	1.421 (2)
C9—C14	1.383 (3)	C21—H21A	0.9600
C9—N2	1.427 (2)	C21—H21B	0.9600
C10—C11	1.383 (3)	C21—H21C	0.9600
C10—H10	0.9300	N1—H1	0.8600
C11—C12	1.369 (4)	N2—N3	1.394 (2)
			
O1—C1—N1	120.55 (15)	C14—C13—H13	119.8
O1—C1—C2	123.12 (16)	C12—C13—H13	119.8
N1—C1—C2	116.32 (15)	C13—C14—C9	118.8 (2)
C3—C2—C7	122.01 (15)	C13—C14—H14	120.6
C3—C2—C1	124.02 (16)	C9—C14—H14	120.6
C7—C2—C1	113.84 (15)	C20—C15—C16	118.08 (16)
C2—C3—C4	116.42 (15)	C20—C15—C3	121.91 (16)
C2—C3—C15	120.98 (16)	C16—C15—C3	119.99 (16)
C4—C3—C15	122.60 (15)	C17—C16—C15	120.78 (17)
C5—C4—C3	119.12 (16)	C17—C16—H16	119.6
C5—C4—C6	103.83 (15)	C15—C16—H16	119.6
C3—C4—C6	136.83 (16)	C16—C17—C18	120.81 (18)
N2—C5—N1	127.97 (15)	C16—C17—H17	119.6
N2—C5—C4	108.43 (15)	C18—C17—H17	119.6
N1—C5—C4	123.53 (16)	O2—C18—C17	114.81 (17)
N3—C6—C4	111.18 (15)	O2—C18—C19	125.76 (17)
N3—C6—C8	118.63 (17)	C17—C18—C19	119.43 (16)
C4—C6—C8	130.17 (17)	C18—C19—C20	119.69 (17)
N4—C7—C2	178.0 (2)	C18—C19—H19	120.2
C6—C8—H8A	109.5	C20—C19—H19	120.2
C6—C8—H8B	109.5	C19—C20—C15	121.20 (18)
H8A—C8—H8B	109.5	C19—C20—H20	119.4
C6—C8—H8C	109.5	C15—C20—H20	119.4
H8A—C8—H8C	109.5	O2—C21—H21A	109.5
H8B—C8—H8C	109.5	O2—C21—H21B	109.5
C10—C9—C14	121.36 (18)	H21A—C21—H21B	109.5
C10—C9—N2	120.09 (18)	O2—C21—H21C	109.5
C14—C9—N2	118.54 (18)	H21A—C21—H21C	109.5
C9—C10—C11	118.8 (2)	H21B—C21—H21C	109.5
C9—C10—H10	120.6	C5—N1—C1	120.54 (14)
C11—C10—H10	120.6	C5—N1—H1	119.7
C12—C11—C10	120.4 (2)	C1—N1—H1	119.7
C12—C11—H11	119.8	C5—N2—N3	110.21 (14)
C10—C11—H11	119.8	C5—N2—C9	130.98 (15)
C11—C12—C13	120.2 (2)	N3—N2—C9	118.76 (14)
C11—C12—H12	119.9	C6—N3—N2	106.35 (15)
C13—C12—H12	119.9	C18—O2—C21	118.36 (16)
C14—C13—C12	120.4 (3)		
			
O1—C1—C2—C3	−175.69 (18)	C4—C3—C15—C20	56.1 (2)
N1—C1—C2—C3	2.7 (3)	C2—C3—C15—C16	54.0 (2)
O1—C1—C2—C7	0.3 (3)	C4—C3—C15—C16	−125.4 (2)
N1—C1—C2—C7	178.62 (16)	C20—C15—C16—C17	−1.0 (3)
C7—C2—C3—C4	−178.28 (17)	C3—C15—C16—C17	−179.57 (17)
C1—C2—C3—C4	−2.6 (3)	C15—C16—C17—C18	0.0 (3)
C7—C2—C3—C15	2.3 (3)	C16—C17—C18—O2	−179.12 (17)
C1—C2—C3—C15	177.93 (17)	C16—C17—C18—C19	1.1 (3)
C2—C3—C4—C5	0.8 (2)	O2—C18—C19—C20	179.00 (17)
C15—C3—C4—C5	−179.76 (16)	C17—C18—C19—C20	−1.3 (3)
C2—C3—C4—C6	−172.7 (2)	C18—C19—C20—C15	0.3 (3)
C15—C3—C4—C6	6.7 (3)	C16—C15—C20—C19	0.8 (3)
C3—C4—C5—N2	−176.16 (16)	C3—C15—C20—C19	179.38 (16)
C6—C4—C5—N2	−0.7 (2)	N2—C5—N1—C1	175.58 (17)
C3—C4—C5—N1	0.9 (3)	C4—C5—N1—C1	−0.9 (3)
C6—C4—C5—N1	176.38 (16)	O1—C1—N1—C5	177.60 (16)
C5—C4—C6—N3	0.4 (2)	C2—C1—N1—C5	−0.8 (2)
C3—C4—C6—N3	174.6 (2)	N1—C5—N2—N3	−176.14 (17)
C5—C4—C6—C8	−177.7 (2)	C4—C5—N2—N3	0.8 (2)
C3—C4—C6—C8	−3.5 (4)	N1—C5—N2—C9	1.4 (3)
C3—C2—C7—N4	171 (8)	C4—C5—N2—C9	178.32 (18)
C1—C2—C7—N4	−5(8)	C10—C9—N2—C5	44.7 (3)
C14—C9—C10—C11	0.3 (3)	C14—C9—N2—C5	−136.3 (2)
N2—C9—C10—C11	179.32 (18)	C10—C9—N2—N3	−137.97 (19)
C9—C10—C11—C12	−1.3 (3)	C14—C9—N2—N3	41.1 (3)
C10—C11—C12—C13	1.4 (4)	C4—C6—N3—N2	0.0 (2)
C11—C12—C13—C14	−0.3 (4)	C8—C6—N3—N2	178.40 (17)
C12—C13—C14—C9	−0.7 (4)	C5—N2—N3—C6	−0.5 (2)
C10—C9—C14—C13	0.7 (3)	C9—N2—N3—C6	−178.39 (17)
N2—C9—C14—C13	−178.32 (19)	C17—C18—O2—C21	177.44 (17)
C2—C3—C15—C20	−124.5 (2)	C19—C18—O2—C21	−2.8 (3)

### Hydrogen-bond geometry (Å, °)

**Table:**

*D*—H···*A*	*D*—H	H···*A*	*D*···*A*	*D*—H···*A*
N1—H1···O1^i^	0.86	2.06	2.8523 (18)	153.

Symmetry codes: (i) −*x*, −*y*+1, −*z*+2.
